# A review of artificial intelligence-based brain age estimation and its applications for related diseases

**DOI:** 10.1093/bfgp/elae042

**Published:** 2024-10-22

**Authors:** Mohamed Azzam, Ziyang Xu, Ruobing Liu, Lie Li, Kah Meng Soh, Kishore B Challagundla, Shibiao Wan, Jieqiong Wang

**Affiliations:** Department of Neurological Sciences, College of Medicine, University of Nebraska Medical Center, Omaha, NE 68198, United States; Department of Computer Science and Engineering, Faculty of Electronic Engineering, Menoufia University, Menouf 32952, Egypt; Department of Neurological Sciences, College of Medicine, University of Nebraska Medical Center, Omaha, NE 68198, United States; Department of Neurological Sciences, College of Medicine, University of Nebraska Medical Center, Omaha, NE 68198, United States; Department of Neurological Sciences, College of Medicine, University of Nebraska Medical Center, Omaha, NE 68198, United States; Department of Biostatistics, College of Public Health, University of Nebraska Medical Center, Omaha, NE 68198, United States; Department of Neurological Sciences, College of Medicine, University of Nebraska Medical Center, Omaha, NE 68198, United States; Department of Genetics, Cell Biology and Anatomy, College of Medicine, University of Nebraska Medical Center, Omaha, NE 68198, United States; Department of Neurological Sciences, College of Medicine, University of Nebraska Medical Center, Omaha, NE 68198, United States

**Keywords:** brain age, biological age, disease diagnosis, machine learning, deep learning

## Abstract

The study of brain age has emerged over the past decade, aiming to estimate a person’s age based on brain imaging scans. Ideally, predicted brain age should match chronological age in healthy individuals. However, brain structure and function change in the presence of brain-related diseases. Consequently, brain age also changes in affected individuals, making the brain age gap (BAG)—the difference between brain age and chronological age—a potential biomarker for brain health, early screening, and identifying age-related cognitive decline and disorders. With the recent successes of artificial intelligence in healthcare, it is essential to track the latest advancements and highlight promising directions. This review paper presents recent machine learning techniques used in brain age estimation (BAE) studies. Typically, BAE models involve developing a machine learning regression model to capture age-related variations in brain structure from imaging scans of healthy individuals and automatically predict brain age for new subjects. The process also involves estimating BAG as a measure of brain health. While we discuss recent clinical applications of BAE methods, we also review studies of biological age that can be integrated into BAE research. Finally, we point out the current limitations of BAE’s studies.

## Introduction

With the significant social change in the current century due to the population aging, our society sectors, especially healthcare, are adversely affected by the associated functional declines and diseases, such as cancer, cardiovascular disease, diabetes, and dementia [1, 2]. Aging is generally described as a gradual accumulator of biological changes in a human subject leading to the progressive decline of various physiological and organ functions [3]. In order to provide adequate care and treatment, it became urgent to study the link between biological aging and potential diseases. Usually, biological age can be estimated from mediums, such as imaging data of an organ (brain) or blood samples [DNA methylation (DNAm)], or physical and functional assessments (grip strength and lung capacity) [4, 5].

Brain age estimation (BAE) stands out among biological age estimation options. This is because recent research studies have shown great success in accurately estimating the brain age of healthy subjects given the neuroimaging data [6, 7]. Brain aging is always accompanied by interactions and specific morphological changes in brain structure across the lifespan. Previous studies have demonstrated that brain structure and function are altered in neurodegenerative diseases, such as Parkinson’s disease (PD) and Alzheimer’s disease (AD) [8]. BAE typically applies machine learning regression algorithms on given neuroimaging data, such as T1-weighted imaging, diffusion-wighted imaging (DWI), and functional magnetic resonance imaging (fMRI), etc., to accurately predict brain ages for new unseen brain imaging. The brain age gap (BAG) is computed as the difference between the predicted brain age $\hat{Y}$ from BAE models and the chronological age $Y$ (i.e. the actual age of human subjects). Mathematically, $BAG = \hat{Y} - Y$, where positive BAG values imply “accelerated” aging, and negative BAG values reflect “delayed” aging.

BAE models tend to learn a reference curve for healthy aging subjects. This allows the model to be a biomarker of brain health by reporting zero or small BAG values for healthy subjects and large BAG values for subjects with brain-related diseases. Such biomarkers are imperative in clinical applications for early detection of diseases, risk assessment, and accurate evaluation of potential treatments [8]. However, the results of BAE models still need to be more reliable due to multiple factors: (1) lack of specificity, (2) bias to gender, race, and scannning devices, (3) limited dataset size, and (4) lack of accounting in BAE studies to environmental factors and genetics that may manipulate human aging. Another challenge is that current BAE models only estimate brain age for input subjects at a specific time point instead of estimating the aging rate across subjects’ lifespans. This makes BAE models fail to meet one of the main criteria established by the American Federation for Aging Research for qualifying aging biomarkers [5, 9].

In this review paper, we discuss the process of BAE in Section 2. Then, we address the potential clinical applications of BAE as a biomarker in Section 3. Next, other approaches for estimating the biological age are presented in Section 4. After that, the current limitations of recent studies in BAE are addressed in Section 5. Finally, we report our conclusions in Section 6.

## Brain age estimation

### System design

Generally speaking, building the BAE model consists of two steps. The first step is the training step, which basically involves training neuroimaging data to learn the model parameters. The second step is testing and involves testing neuroimaging data for model evaluation. The testing data are regularly distinct from the training data and are often constrained to follow the same training data distribution.

**Figure 1:**
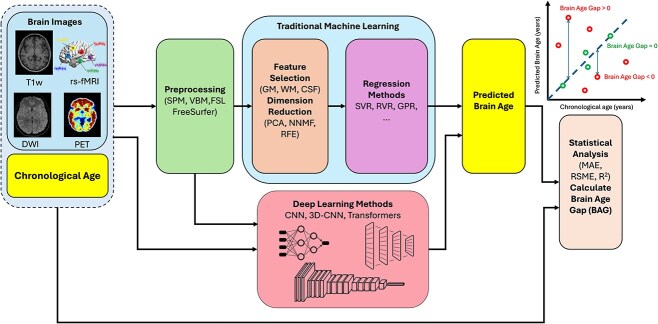
The BAE pipeline involves preprocessing brain images to enhance critical information, followed by feature extraction and prediction using traditional regression (e.g., SVR) or deep learning (e.g., CNNs), with BAG computed as the difference between predicted and chronological age.

Figure 1 illustrates the pipeline of the BAE process. The process starts by collecting neuroimaging data from healthy control (HC) subjects, along with the corresponding age, known as chronological age. The collected data are further split into training and testing data. In the BAE training stage, we first preprocess the input data to eliminate noise and highlight the critical details. Then, we typically apply machine learning to the processed input.

Machine learning methods are categorized into two types: traditional machine learning methods and deep learning methods. Traditional machine learning methods require feature engineering, which extracts specific features and performs feature selection/reduction. After that, the engineered features are fed into one of the traditional regression methods to learn the model parameters and predict the brain age. If deep learning methods are used in BAE, they can be either directly applied to the raw input data or the processed input [10]. The difference between the predicted age and the corresponding chronological age is used to update model parameters in the training stage, calculate the BAG, and perform some statistical analysis in the testing stage. It is worth noting that the testing stage in machine learning generally does not involve updating the model parameters. In some studies [11], the BAE process may involve a validation step after the training step and before performing the final model evaluation in the testing step. This validation step often involves one of these strategies: k-fold cross-validation or bootstrapping [4].

### Input neuroimaging data modalities

Neuroimaging is regarded as a noninvasive method that captures the whole brain of human subjects, including microstructural and morphological features. Magnetic resonance imaging (MRI) modalities usually capture information about the anatomy of the brain and have the following types: (i) T1-weighted (T1w), which shows desirable performance in BAE; (ii) T2-weighted (T2w); (iii) T2-FLAIR; (iv) Task-functional MRI (t-fMRI); and (v) Resting state-functional MRI (rs-fMRI). Some other modalities exist, such as positron emission tomography (PET) and diffusion tensor imaging (DTI). The reader can get more information about these modalities in this recent review [32]. Table 1 lists the standard brain imaging datasets that appeared in the recent studies of BAE.

**Table 1:** List of popular datasets used for brain age prediction

No.	Dataset	HC(#)	Age	Imaging	Genetics
1	IXI [12]	$593$	20–86	T1w, DTI	
2	ADHD-200 [13]	$500$	7–21	T1w, rs-fMRI	
3	ADNI [14]	$923$	40–100	T1W, rs-fMRI,PET, DTI	✓
4	PPMI [15]	$393$	30–89	T1w, DTI	✓
5	UK-BB [16]	$\sim $ $40\,000$	40–69	T1w, DTI, rs-fMRI, ASL	✓
6	OASIS1 [17]	$316$	18-96	T1w	
7	OASIS2 [17]	$72$	60–96	T1w	
8	OASIS3 [17]	$755$	42–95	T1w, DTI, rs-fMRI, ASL	
10	CAM-CAN [18]	$653$	18–88	T1w, rs-fMRI, MEG	
11	HCP-D [19]	$652$	5–21	T1w, T2w, rs-fMRI, t-fMIR, DTI, ASL	
12	HCP-YA [20]	$1003$	22–37	T1w, rs-fMRI	
13	HCP-A [21]	$725$	36–100	T1w, T2w, rs-fMRI, t-fMRI, DTI,ASL	
14	DLBS [22]	$350$	20–89	T1w, PET	✓
15	BNU [23]	$180$	17–25	T1w, rs-fMRI, DTI	
16	ABIDE I [24]	$573$	7–64	T1w, rs-fMRI	
17	ABIDE II [25]	$593$	5–64	T1w, rs-fMRI, DTI	
18	SALD [26]	$494$	19–80	T1w, rs-fMRI	
19	PNC [27]	$1601$	8–21	T1w, rs-fMRI, DTI, ASL	✓
20	ABCD [28]	$11\,892$	8–20	T1w, rs-fMRI, t-fMRI, DTI, ASL	✓
21	OpenBHB [29]	$5330$	7–86	T1w, T2w, DWI, rsfMRI	
22	CC-359 [30]	$359$	29–80	T1w	
23	NCANDA [31]	$831$	12–21	T1w, rs-fMRI, DTI	

HC: Healthy Control subjects

### Preprocessing tools

Preprocessing is an essential part of BAE that takes the raw imaging data as an input and conducts the lowermost level of abstraction to pinpoint the important details and filter out the distortions for the downstream analysis [33]. MRI preprocessing usually involves spatial normalization for mapping all scans to a template scan, resampling for minimizing variations between the reference scan and the following scans, and spatial smoothing for unifying the voxel size or scan space [34, 35].

Popular MRI preprocessing tools are the following: (i) Freesurfer [36] is a region-based software for extracting cortical and subcortical measurements, such as surface area, volumes, and thickness values; (ii) Statistical Parametric Mapping [35] uses a voxel-based method to segment the input MRI image into the following regions: gray matter (GM), white matter (WM), and cerebrospinal fluid (CSF); (iii) FSL [37] is a software that offers two types of interfaces: a command line and graphical user interface. The software contains several analysis tools for several data modalities, including DTI, MRI, and fMRI. It is also used for motion correction, registration, and brain extraction.

### Feature selection/reduction

In this step, the BAE process includes feature selection and/or reduction strategies. In feature selection, we usually keep the most essential and relevant features for BAE and exclude the remaining features. Features reduction (a.k.a dimension reduction) creates a new smaller feature set than the initially extracted features. Principal component analysis [38] represents one of the most popular techniques in feature reduction using linearly uncorrelated features called principal components.

### Machine learning methods

BAE models mainly rely on machine learning regression methods for age prediction. The models are often trained in supervised mode (i.e. valid access to chronological age) on brain imaging data of HC subjects to build a reference curve based on brain structures. This turns BAE models to be used as clinical biomarkers for brain health and early detection of age-related diseases, such as AD and PD. Check Section 3 for more details about the clinical applications of BAE models.

Machine learning methods, which aim to learn a specific task by capturing patterns from training data that relate inputs (e.g. neuroimaging data) to the target outputs (e.g. predicted age), can be categorized based on the input brain imaging data: voxel-based, surface-based, and pixel-based. Recently, voxel-based methods have shown promising results in capturing the different aging rates in multiple brain regions [39]. However, these methods are hindered by the limited voxel resolution [4]. Surface-based methods use a triangled mesh representation of GM, WM, and CSF regions. In pixel-based methods, there are three strategies for training the model: whole slices [4], some slices [40], or the most significant slices [41].

Machine learning methods can also be categorized into two types based on the learning strategy. The first type is called traditional machine learning methods. The second type is called deep learning methods. Table 2 contains the results of recent and popular BAE methods, including both types of machine learning techniques.

**Table 2:** Results of BAE studies on healthy individuals. Studies that adopt only local BAE or merge it with global BAE have $\checkmark $ in the “local” column.

	Local	Subjects	Age	Model	Modality	Results			
						MAE	RMSE	$R^{2}$	$\rho $
Traditional machine learning									
Cole *et al*. [10]		$2001$	18–90	GPR	T1w	$4.41$	$5.43$	$0.91$	–
Aycheh *et al* [42]		$2911$	45–91	GPR	T1w	$4.05$	$5.16$	–	–
Beheshti *et al*. [43]		$100$	19–61	3D Patch [44] + SVR	T1w	$1.66$	$3.00$	$0.94$	–
Niu *et al*. [45]		$839$	8–21	Ridge Regression	T1w, rs-fMRI, DTI	$1.41$	–	$0.766$	–
Niu *et al*. [45]		$839$	8–21	SVR	T1w, rs-fMRI, DTI	$1.43$	–	$0.756$	–
Niu *et al*. [45]		$839$	8–21	GPR	T1w, rs-fMRI, DTI	$1.38$	–	$0.774$	–
Cherubini *et al*. [46]	✓	$120$	20–74	LR	T1w, T2w, DTI, FLAIR	–	–	$>0.4$	$0.963$
Kaufmann *et al*. [47]	✓	$18\,990$ female	3–96	XGBoost	T1w	–	–	–	0.93
Kaufmann *et al*. [47]	✓	$16\,484$ male	3–96	XGBoost	T1w	–	–	–	0.94
De Lange *et al*. [48]		$18\,578$ (UKB)	45–82	XGBoost	T1w	$4.18$	$5.2$	$0.521$	–
De Lange *et al*. [48]		$311$ (CAM-CAN)	18–87	XGBoost	T1w	$6.78$	$8.43$	$0.79$	–
Baecker *et al* [11]		$10\,824$	47–73	RVR	T1w	$3.66$	$4.51$	$0.53$	–
Basodi *et al*. [49]		$1591$	8–21	Decenteralized SVR	T1W (GM)	$3.1$	$3.6$	–	–
Luo *et al* [50]		$1101$	12–82	Ensemble of 3 Models	rs-fMRI	$7.73$	$9.765$	$0.6$	–
Han *et al* [51]		$125$	12–85	XGBoost	rs-fMRI	$5.14$	$6.16$	–	$0.87$
Guan *et al* [52]		$505$	6–85	PLSR	T1w, rs-fMRI	$8.24$	–	–	$0.86$
Ganaie *et al* [53]		$788$	18–94	Twin SVR	T1w	$2.77$	$3.56$	$0.97$	–
Deep learning									
Cole *et al*. [10]		$2001$	18–90	3DCNN	Raw T1w	$4.65$	$6.46$	$0.88$	–
Niu *et al*. [45]		$839$	8–21	DNN	T1w, rs-fMRI, DTI	$1.38$	–	$0.753$	–
Pardakhti & Sajedi [54]		$562$	20–86	3D-CNN	T1w	$5.15$	$13.497$	–	–
Poloni & Ferrari [55]		$774$	20–70	EfficientNet	T1w (3D patches)	$3.64$	$5.32$	–	$0.94$
Popescu *et al*. [56]	✓	$3463$	18–90	U-Net	T1w	$9.94$	–	–	0.75
He, Feng *et al*. [6]		$6049$	0–97	CNN + Transformer	T1w	$2.38$	–	–	$0.988$
He, Grant *et al*. [7]		$8379$	0–97	CNN + Transformer	T1w	$2.7$	–	–	$0.985$
Wang *et al*. [57]		$2406$	17–60	3DCNN	DTI	$2.79$	–	–	$0.93$
Gianchan *et al*. [39]	✓	$651$	18–88	U-Net	T1w	$5.3$	–	–	–

#### Traditional machine learning methods

Traditional machine learning methods refer to machine learning methods requiring hand-crafted features as input. Since BAE models are usually regression models designed for predicting real numbers instead of a finite number of categories, we draw down our discussion for the traditional regression methods. Support vector regression (SVR), linear regression (LR), relevance vector regression (RVR), and Gaussian process regression (GPR) represent famous example methods that are adopted in several BAE models [10, 11, 45, 58].

LR represents the most straightforward and understandable regression method. Both SVR and RVR are designed to capture nonlinear interactions in learning. Since SVR learns multivariate prediction rules from one example and generalizes it to distinct examples, it can provide unbiased age prediction. However, SVR requires additional training for parameter optimization [59]. Unlike SVR, RVR shows more robustness to different scanners and captures changes related to the whole brain [60]. While the methods above are considered parametric methods, GPR is considered a nonparametric method that applies multivariate Gaussian distribution over an infinite number of variables. This allows GPR to model nonlinear relationships and show more flexibility compared with the previous parametric methods [10].

As reported in Table 2, several BAE strategies adopted the abovementioned machine learning algorithms. Specifically, both Cole *et al*. [10] and Aycheh *et al*. [42] applied the GPR algorithm to the extracted features from T1w imaging input. Although Aycheh *et al*. [42] reported better results, this may be attributed to the reduced age range and increased number of subjects. Beheshti *et al*. [43] coupled 3D patch-based grading with the SVR algorithm to estimate the brain age score. Learning from multi-modal imaging features was extensively explored in this study [45]. The authors also compared the results of using three different traditional algorithms (ridge regression, SVR, and GPR) and concluded that multi-modal features are beneficial in brain age prediction.

De Lange *et al*. [48] also adopted the XGBoost algorithm [61] and studied two distinct T1w datasets. They also accounted for age bias correction to enhance the model’s generalizability. Interestingly, Baecker *et al*. [11] compared three machine learning models (i.e. SVR, RVR, and GPR) under two formats of T1w inputs: voxel-based and region-based. They also explored the feasibility of conduction dimensionality reduction with voxel-based models. At the end of their study, a decision tree is reported to guide researchers in designing their BAE studies. Federated learning was explored in a BAE study by Basodi *et al*. [49]. Specifically, decentralized SVR is adopted to learn model parameters from multiple sites without sharing the data among sites. However, this study is limited by the narrow age range considered. Unlike previous works, which mainly used T1w inputs, Luo *et al*. [50] and Han *et al*. [51] used rs-fMRI instead. Luo *et al*. [50] focused on performing ensemble learning of three learning models: ridge regression, Bayesian ridge [62], and elastic net [63]. On the other hand, Han *et al*. [51] compared the performance of six different learning algorithms and concluded that XGBoost exhibits superior performance. Recently, Guan *et al*. [52] investigated the performance of the learning algorithm partial least squares regression (PLSR) [64] on four T1w features (i.e. cortical thickness, GM volume, mean curvature, and surface area) and three rs-fMRI features (i.e. fALFF, ReHo, and ALFF). They concluded that (i) the cortical thickness feature is extremely vital in age prediction, and (ii) the left hemisphere contributes more to age prediction. Finally, Ganaie *et al*. [53] demonstrated outstanding performance by applying an improved version of the twin SVR algorithm [65] to input MRI features.

#### Deep learning methods

Deep learning methods generally refer to the machine learning approaches that adopt neural networks with several layers. These methods do not require feature engineering and automatically extract features from the input data. They also enjoy the high capacity of capturing interactive linkage among features during training. This allows deep learning models to successfully handle complex computer vision tasks such as image segmentation and object detection [6]. The most famous architecture in deep learning is a convolutional neural network (CNN) [66]. Transformer [67] is also a famous architecture that was initially used in natural language processing and has recently become popular in visual recognition tasks [68].

In BAE studies, popular deep learning MRI-based methods are Age-Net [69], DeepBrainNet [70], deep relation learning [6], and global-local BAE [7]. As illustrated in Table 2, Cole *et al*. [10], and Niu *et al*. [45] compared the performance of deep learning methods with the traditional learning algorithms. Both studies indicated similar results of learning methods, highlighting potential improvement in deep learning algorithms, given the availability of more training samples and more computational resources to train larger models. Furthermore, Paradakhi & Sajadi deployed 3D-CNN on T1w input data and investigated the performance of traditional algorithms on the output features from the deep model compared with the fully connected layers. Instead of using MRI input data, Wang *et al*. [57] used DTI data in training 3D-CNN and reported outstanding results in age prediction. While most of the previous methods mainly adopt CNN in their framework, He *et al*. [7] adopt transformers [67], along with fusing extracted features from local patches as well as the whole image of T1w input.

#### Local BAE

Despite the increasing number of studies on brain age [6, 53, 57, 71], most research treats the brain as a whole and predicts brain age on a global level. The brain, however, exhibits mosaic aging, wherein different regions may age at varying rates or undergo distinct age-related changes. Compared with global brain age, a finer-grained approach may better reveal the spatial pattern of BAG in various diseases and track brain changes over time as diseases progress [56]. Limited work on local BAE exists. Cherubini *et al*. [46] built LR models for each voxel using features from voxel-based morphometry and DTI. Kaufmann *et al*. [47] trained separate models for each brain region, providing local or regional information but limiting the incorporation of contextual and global information. Popescu *et al*. [56] were the first to employ deep learning for BAE. Using the U-Net architecture, the researchers produced individualized 3D maps of brain-predicted age and demonstrated distinct local brain-age patterns in individuals with mild cognitive impairment (MCI) or dementia. This study clearly shows that local BAE has the potential to provide spatial information, leading to a more mechanistic understanding of individual differences in brain aging patterns in health and disease. Finally, Gianchandani *et al.* [39] deployed multi-task deep learning model on T1w images to predict age at the voxel level and global level. They also performed three types of brain segmentation on GM, WM, and CSF regions.

#### Model selection

According to the no free lunch theorems [72], no identical algorithm is the best for all the machine learning tasks. Hereby, there is no identical machine learning algorithm that is the best in all BAE problems. Factors such as dataset size, input data modality, noise ratio in data, data distribution, gender, and task complexity play an imperative role when deciding to adopt a specific machine learning algorithm.

Traditional machine learning methods are easier to explain and interpret than deep learning methods due to their simple design. This enables the identification of the important features and their impact on the overall performance. However, these methods require many engineering efforts to extract features from the input. The hand-crafted features can sometimes be restrictive, resulting in the omission of crucial features in the raw input. On the other side, deep learning methods are more robust against bias and have the potential to generate desirable insights directly from the neuroimaging input, even without conducting preprocessing [10]. This, of course, obviates the need to consume hours of preprocessing neuroimaging inputs and enhances the clinical applicability of BAE models [6].

Some studies in BAE adopt multiple models to enhance age prediction accuracy. They usually adopt ensemble learning to train multiple machine learning models in age prediction tasks. Boosting, bagging, and stacking are the broad categories of ensemble learning [73].

### Model evaluation and statistical analysis

Table 3 reports the prevalent metrics for assessing the accuracy of BAE models. The most widely used metric is mean absolute error (MAE), which computes average absolute BAG across subjects. However, MAE is susceptible to the age range of the test set, leading to inaccuracies in cross-study comparisons [39]. To obtain a more holistic assessment of models performance, recent studies usually report $R^{2}$ metric and use a violin plot. F-statistics and cumulative score are also reported in recent studies [6, 7].

**Table 3:** Common evaluation metrics for machine learning regression models, such as brain age prediction models

Metric	Equation
*MAE*: average of absolute residuals ${\downarrow }$	$\frac{1}{N} \sum ^{N}_{i=1} \mid \hat{Y_{i}} - Y_{i}\mid $
*RMSE*: $\sqrt{\mathrm{squared}\quad \mathrm{residuals}}$ ${\downarrow }$	$\sqrt{\frac{1}{N} \sum ^{N}_{i=1}(\hat{Y_{i}} - Y_{i})^{2}}$
* $R^{2}$ *: variance in $\hat{Y}$ explained by $Y$ ${\uparrow }$	$1 - \frac{\sum ^{N}_{i=1}(\hat{Y_{i}} - Y_{i})^{2}}{\sum ^{N}_{i=1}(Y_{i} - \overline{Y_{i}})^{2}}$
* $\rho $ *: Pearson’s correlation coefficient ${\uparrow }$	$\frac{\sum ^{N}_{i=1}(\hat{Y_{i}} - \overline{\hat{Y_{i}}}) (Y_{i} - \overline{Y_{i}})} {\sqrt{{\sum ^{N}_{i=1}(\hat{Y_{i}} - \overline{\hat{Y_{i}}})}^{2}} \sqrt{{\sum ^{N}_{i=1}(Y_{i} - \overline{Y_{i}})}^{2}}}$

$Y_{i}$ : true value of input sample $i$. $\hat{Y}_{i}$: predicted value of input sample $i$. $N$: number of samples. $\overline{Y}$: $\frac{1}{N}\sum ^{N}_{i=1}Y_{i}$. $\overline{\hat{Y}}$: $\frac{1}{N}\sum ^{N}_{i=1}\hat{Y_{i}}$. ${\downarrow }$: lower value shows better fit. ${\uparrow }$: higher value shows better fit.

Although there are several works in BAE studies, most of them lack standard evaluation protocols. Specifically, many aspects need to be addressed in model evaluation, including (i) model generalizability from one site to new sites, (ii) reliability of estimated ages on repeated measurements, (iii) standard age distribution of the testing data, and (iv) estimated age consistency on longitudinal subjects. More *et al*. [74] and Dular *et al*. [75] have recently paved the way by providing standard protocols to evaluate BAE models. While More *et al*. [74] mainly focused on works that adopt traditional machine learning algorithms, Dular *et al*. [75] paid more attention to deep learning-based techniques. Also, integrating domain adaptation strategies to BAE design can contribute to generalizability on multi-site data, given its great success in recent works [76–78].

### Bias correction to BAE

Despite considerable efforts to reduce the prediction error in BAE models, a systematic bias persists when predicting individual brain ages. This bias typically manifests as overestimation in younger subjects and underestimation in older subjects. The causes of this bias vary depending on the type of brain age model. In linear models, the bias arises due to the orthogonality between predicted brain age and BAG, which restricts the angle between them to values between 0 and 90 deg [79]. In contrast, for nonlinear models, the bias is thought to stem from regression dilution, which is linked to the non-Gaussian distribution of chronological age [80–82].

Bias correction is critical because BAG is intended to serve as an informative index of individual brain health. Previous studies have shown that higher order correction methods, such as quadratic corrections, yield similar results to linear correction methods [82]. As a result, linear correction methods are widely adopted in most BAE studies [74, 83].

Bias correction strategies can be categorized into two types: sample-level and age-level corrections (see Table 4). Sample-level bias correction, which adjusts the BAG bias across all samples, has been used for several years. The three main linear correction methods include Cole’s method [84], de Lange’s method [48], and Beheshti’s method [85], with Beheshti’s method shown to be equivalent to de Lange’s. These methods effectively reduce the overall BAG bias, bringing the mean BAG across all samples closer to zero. However, they fail to address the bias observed in samples of the same chronological age, known as age-level bias [86]. To resolve this issue, an age-level bias correction method was recently proposed [86]. This approach is recommended as a follow-up step after applying sample-level correction to ensure both types of bias are adequately corrected.

**Table 4:** Bias correction methods in BAE studies

Authors	Strategy	Equation
Cole *et al*. [84]	Sample-level	$\epsilon _{c} = \frac{(\hat{Y} - \beta _{1})}{\alpha _{1}} - Y$
De lang *et al*. [87]		$\hat{\epsilon } = \alpha _{2} \times Y + \beta _{2}$
		$\epsilon _{c} = \hat{Y} - (\alpha _{2} \times Y - \beta _{2})$
Beheshti *et al*. [85]		$\epsilon = \alpha _{2} \times Y + \beta _{2}$
		$\epsilon _{c} = \hat{Y} - [(\alpha _{2} + 1) \times Y + \beta _{2}]$
Zhang *et al*. [86]	Age-level	$\epsilon ^{ia}_{c} = \frac{(\epsilon _{i} - \mu _{i})}{\alpha _{a}}$

$Y$ : chronological age. $\hat{Y}$: predicted brain age, $\hat{Y} = \alpha _{1} \times Y + \beta _{1}$. $\alpha _{1}$: slope of regressing $\hat{Y}$ on $Y$. $\beta _{1}$: intercept of regressing $\hat{Y}$ on $Y$. $\hat{\epsilon }$: predicted age difference. $\epsilon $: calculated age difference, $\epsilon = \hat{Y} - Y$. $\epsilon _{c}$: corrected age difference. $\epsilon ^{ia}_{c}$: corrected age difference for sample $i$ at age $a$. $\alpha _{2}$: slope of regressing $\epsilon $ on $Y$. $\beta _{2}$: intercept of regressing $\epsilon $ on $Y$. $\mu _{a}$: mean age difference $\epsilon $ at age $a$. $\sigma _{a}$: standard deviation of age difference at age $a$.

## Clinical applications to BAE

### BAG as a biomarker for brain health and disease diagnosis

The application of BAG has significant potential in clinical settings, particularly for diagnosing, prognosing, and making treatment decisions. A positive BAG, where an individual’s brain appears older than their chronological age, is commonly associated with neurodegenerative diseases such as AD and MCI. Studies using MRI and PET imaging have demonstrated that a significant BAG can predict cognitive decline and disease progression in these conditions, making it a valuable tool for early detection and monitoring. In contrast, smaller BAGs may be observed in other conditions, reflecting less severe or different types of brain changes. This variability in BAG across different diseases highlights its utility in differential diagnosis and in tailoring treatment strategies based on the severity of brain aging observed [8].

**Figure 2:**
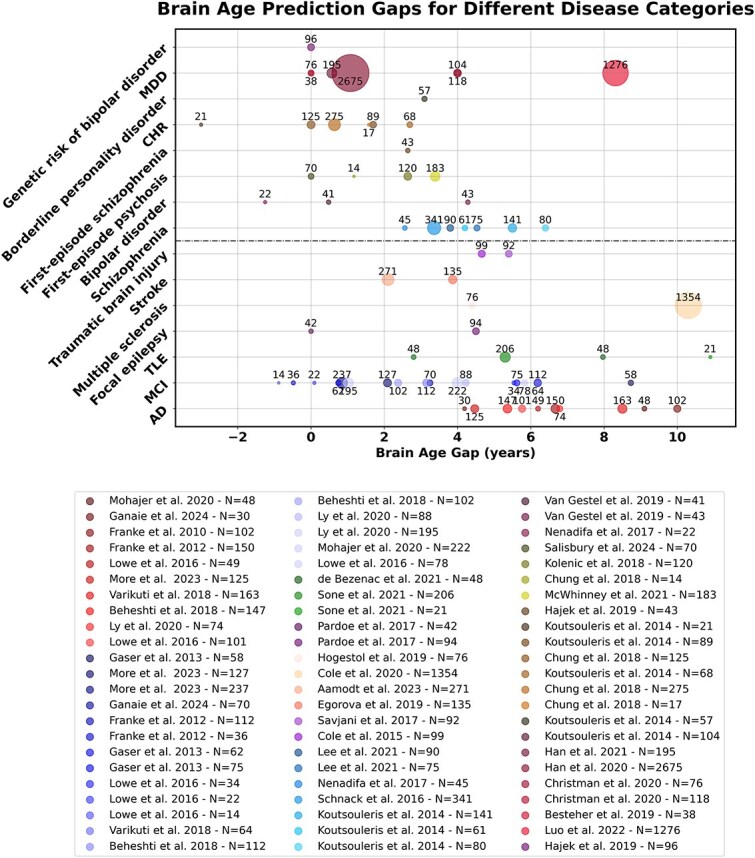
BAE studies on common neurological disorders and psychiatric disorders. Each study is denoted as a circle, and the circle size increases proportionally with the number of incorporated subjects $N$.

Brain age studies extend beyond AD and MCI, encompassing a range of clinical populations including those with traumatic brain injury, multiple sclerosis (MS), stroke, and psychiatric disorders such as schizophrenia, bipolar disorder, and major depressive disorder. A notable pattern is the consistently significant BAG observed in individuals with schizophrenia, which aids in differential diagnosis and identifies those at greater risk for severe disease progression. This differential diagnostic capability is crucial, as it helps distinguish between overlapping symptoms of various psychiatric disorders. For instance, while schizophrenia often shows a high BAG, bipolar disorder’s effect on BAG is less consistent, highlighting the importance of BAG in fine-tuning diagnostic processes [88]. Figure 2 plots the results of recent BAE studies on patients with the corresponding BAG values.

Furthermore, BAG serves as a biomarker of overall brain health. A higher BAG correlates with various markers of poor health, such as cognitive decline, reduced physical strength, and slower walking speed. Conversely, lifestyle factors like regular meditation, music practice, and physical activity are associated with lower BAGs, indicating protective effects. This makes BAE a valuable tool for early disease detection, offering a biological dimension to the screening process and potentially enabling earlier and more accurate diagnoses. Longitudinal studies suggest that BAG monitoring could predict treatment responses, allowing for more personalized and effective management of conditions ranging from neurodegenerative diseases to psychiatric disorders. This evidence underscores the suitability of BAE for clinical applications, supporting its integration into routine clinical practice for enhanced patient care [89].

### Genetic heterogeneity of BAG

Recent advancements in brain imaging and genetics have shed light on brain age as a biomarker for understanding the aging process and its genetic underpinnings. Multiple studies have explored how genetic variants influence brain aging.

Kim *et al*. [90] conducted an in-depth study using multimodal examples from the UK-BB dataset, including brain MRI, genomics, blood-based biomarkers, and metabolomics, to investigate genetic variants associated with BAG. Throughout the genome-wide association (GWAS) and Mendelian randomization (MR) analysis, they found genetic variants in KLF3-AS1 and STX1 regions and revealed a causal connection between immune-related biomarkers and BAG, indicating a genetic and immune link to brain aging. Leonardsen *et al*. [91] used neural networks with genetic architecture to estimate brain age in a cohort of over $53\,000$ individuals. The GWAS identified eight genomic regions associated with BAG, and the MR analysis demonstrated causal relationships between BAG and neurological disorders, such as AD and bipolar disorder. Similarly, Wen *et al*. [92] leveraged multimodal brain imaging and genomic data to investigate the genetic architecture of brain aging. Their study confirmed the polygenic nature of brain aging and identified eight genomic regions associated with BAG, echoing the findings of Leonardsen *et al*. [91]. Their results highlighted the genetic links between neuropsychiatric disorders and accelerated brain aging.

Ning *et al*. [93] investigated the associations between environmental and genetic factors on brain aging using UK-BB data. The study revealed that brain age is influenced by lifestyle factors, such as tobacco and alcohol consumption, and identified a significant association with the MAPT gene. In a tract-based analysis, Salih *et al*. [94] examined how specific WM tracts contribute to brain aging. They discovered that limbic tracts provide the most accurate estimates of brain age and are significantly associated with lifestyle factors and genetic variants. This suggests that variations in WM integrity are influenced by genetic factors and may contribute to accelerated brain aging. Expanding on these genetic studies, Jawinski *et al*. [95] analyzed the heritability of BAG and its relationships with over $1000$ health traits, including mental health (e.g. depression) and physical health (e.g. diabetes). They identified $25$ loci associated with brain aging, with MAPT emerging as a significant locus, reinforcing its relevance to AD and broader brain aging mechanisms. Ning *et al*. [96] employed CNN to improve the accuracy of age predictions. Their study uncovered new genetic loci associated with brain aging, demonstrating that accurate models help detecting genetic factors involved in the aging process.

Overall, the achievement of genetic research underscores the polygenic nature of brain aging and its intricate relationships with lifestyle factors, immune responses, and neuropsychiatric conditions. As machine learning and neuroimaging techniques continue to evolve, they are enabling more precise identification of genetic variants and pathways involved in brain aging, offering valuable insights into the biological mechanisms driving this process.

### Longitudinal BAE studies

While traditional BAE studies typically assess individuals at a single time point, longitudinal BAE studies, which follow the same individuals over time, offer several distinct advantages. Most notably, the longitudinal design enhances the detection of gradual or subtle changes in neuroimaging phenotypes that are often missed in cross-sectional analyses. Additionally, the longitudinal design provides greater statistical power and sensitivity, which are crucial for identifying markers of neurodegenerative diseases [97].

Recent longitudinal studies have demonstrated the potential of BAE in various neurological conditions. For instance, a longitudinal study examining BAG and post-stroke neurocognitive disorder (NCD) found that individuals with lower BAG had a reduced risk of developing post-stroke NCD up to 36 months after a stroke. This finding suggests that a brain that appears younger relative to chronological age is more resilient and associated with milder cognitive impairments following stroke [98]. In the context of AD, longitudinal studies have shown that individuals with non-amnestic (non-memory-related) AD exhibit higher brain BAG scores compared with those with amnestic (memory-related) AD. This highlights the utility of brain BAG in distinguishing between different subtypes of Alzheimer’s and in assessing disease severity [99]. Similarly, changes in brain BAG over time have been linked to disease progression in MS. Specifically, an increasing brain BAG has been associated with worsening disability scores on the Expanded Disability Status Scale, indicating that brain BAG could serve as a valuable biomarker for tracking disease progression in MS patients [100]. Furthermore, in a longitudinal study involving the ABCD cohort, researchers found that as puberty progresses, brain maturation accelerates. Faster pubertal development was linked to slightly accelerated brain growth, suggesting that the timing and pace of puberty may influence brain development, with potential implications for future cognitive and mental health outcomes [101].

A major challenge limiting the progress of longitudinal BAE studies is the scarcity of available longitudinal neuroimaging datasets. Widely used datasets such as ADNI, PPMI, and the ABCD Study provide valuable resources, but their number is limited (see Table 1 for more details). Recently, new longitudinal datasets, including NCANDA and HCP datasets, have been released, offering additional data and expanding opportunities for research in this field.

### Beyond BAG estimation for clinical applications

Although BAG estimation has shown the potential for identifying neurodegenerative diseases, it seems using a single value to summarize the whole regional variations in brains is insufficient. Consequently, the estimated value of BAG lacks spatial specificity, which is imperative in early screening [102]. Therefore, some recent studies have paid more attention to maximizing the gain of the extracted age information in BAE instead of only focusing on enhancing the accuracy of BAG estimation [102, 103].

For example, Ran *et al*. [102] developed brain age vector, comprising regional variations of the input brain imaging with the help of Shapely Additive Explanation [104]. The proposed vector has demonstrated promising results in early disease detection with high accuracy. Sihag *et al*. [103] went even further and additionally accounted for data heterogeneity and the limited size of training data in their study. They resorted to foundation models [105] to robustly interpret brain anatomy for any arbitrary brian atlas, expanding brain age use in numerous biomedical applications. Foundation models [105] have recently emerged in artificial intelligence (AI) to shift models’ deployment from context-specific paradigm with narrow applicability to generic models that can readily be adapted to several downstream tasks with distinct contexts.

## Estimating biological age with omics technologies

Estimating biological age, as opposed to chronological age, offers a more accurate reflection of an individual’s physiological state and their risk for age-related diseases [106, 107]. Recent advancements in high-throughput omics technologies have fueled the development of tools to quantitatively assess biological aging. These include epigenomic, transcriptomic, and proteomic data, which can be integrated with machine learning to create “aging clock” that reveals novel biomarkers of aging. In this section, we first explore studies focused on DNAm age, a widely used epigenetic measure of biological aging. We then review research on transcriptomic and proteomic approaches to estimate biological age. Finally, we discuss the relationship between brain age and other biological measures, providing insight into the integration of brain health with systemic aging processes.

### DNAm age (epigenetic clocks)

Epigenetic clocks (referred to as “DNAm age”), which are predictive models based on DNAm patterns, have emerged as powerful tools to estimate an individual’s biological age. These clocks use patterns of methylation at specific CpG sites across the genome to predict an individual’s biological age [108]. Studies, such as those using Horvath’s clock, have demonstrated that DNAm age is highly correlated with chronological age and can predict various health outcomes more accurately than chronological age alone.

For instance, a study in the ALSPAC cohort derived four methylation age measures in late adolescence and compared them with brain age measures from structural neuroimaging. The results showed that smoking and BMI were associated with advanced methylation age but not brain age, indicating distinct pathways of aging in different tissues and highlighting the independence of these measures in adolescents [109]. DNAm PhenoAge is an advanced DNAm-based biomarker that predicts biological aging and is associated with various health outcomes, including cardiovascular disease and mortality [110]. It is derived using a novel two-step method that trains an epigenetic predictor of phenotypic age, which reflects physiological dysregulation, rather than chronological age. This approach improves predictions over earlier DNAm biomarkers by targeting specific CpG sites, such as those associated with inflammatory markers and oxidative stress pathways.

Research in animal models has also contributed to our understanding of DNAm changes in relation to lifespan. A study on rockfish, known for their exceptional longevity, revealed that certain DNAm changes, such as the transition from CpG to TpG mutations, were more prevalent in species with longer lifespans. While these mutations do not directly cause longevity, they reflect a mutational signature that aligns with species-specific life history traits. Similar patterns in humans have allowed scientists to develop DNAm-based age estimators that surpass chronological age in predicting age-related health risks [111].

The value of DNAm-based clocks is not limited to cross-sectional studies but has increasingly been explored in longitudinal settings to assess how these markers evolve over time. Reynolds *et al*. [112] investigated the genetic and environmental influences on DNAm changes over aging population. Their decade-long study on twins revealed that while genetic contributions to DNAm were stable over time, environmental factors and individual-specific experiences played a greater role in driving DNAm changes during aging. Sites associated with senescence and aging appeared to be more heritable, suggesting a nuanced interplay between genetic predisposition and environmental exposure in shaping DNAm over time. Longitudinal studies also suggest that DNAm changes can capture the systemic nature of biological aging. Research by Joyce *et al*. [113] examined the role of GrimAge, an epigenetic clock designed to predict cardiovascular health and disease risk. This study highlighted how accelerated epigenetic aging is linked to the loss of cardiovascular health, thus linking biological aging processes in the cardiovascular system to broader health outcomes, including brain aging. Further expanding on the systemic implications of DNAm, Pang *et al*. [114] investigated the impact of COVID-19 and messenger RNA vaccination on epigenetic clocks in older individuals. Their findings revealed age-related divergence in DNAm patterns following infection, with older individuals showing significant increases in PhenoAge and GrimAge post-infection. Interestingly, vaccination, particularly with the Moderna vaccine, was found to mitigate this age acceleration, underscoring the potential for immune interventions to influence aging processes. An interesting longitudinal study to explore how environmental stressors can accelerate biological aging, particularly in vulnerable populations, is conducted by Smith *et al*. [115]. They analyzed the relationship between epigenetic age acceleration and posttraumatic stress disorder in women exposed to large-scale disasters. The results revealed that racial minorities (Black and American Indian women) experienced accelerated epigenetic aging, linking environmental stress with accelerated biological aging and subsequent health risks. Lastly, Verschoor *et al*. [116] and Vetter *et al*. [117] explored the relationship between DNAm age and functional capacity (e.g. frailty) on older adults. To sum up, longitudinal studies have shown that DNAm changes over time reflect biological aging across different tissues and conditions. These findings emphasize the complexity of aging and the significance of DNAm-based biomarkers in capturing the influence of genetic, environmental, and lifestyle factors on biological age.

### Transcriptomic and proteomic approaches

Beyond DNAm age, transcriptomic and proteomic data offer additional layers of molecular information. Transcriptomic analysis involves examining the complete set of RNA transcripts produced by the genome under specific circumstances or in a particular cell. Changes in gene expression profiles can indicate aging-related processes and help estimate biological age. Proteomic analysis, which studies the full set of proteins expressed by a genome, provides insights into the functional state of cells and tissues. Proteins are directly involved in most biological processes, and their abundance and modification states can reflect the biological age of an organism. Combining posttranslational products like transcriptomic and proteomic data with machine learning techniques enhances the interpretability and experimental testability of biological age estimation models and uncovers functional gene networks associated with aging [106].

Martínez-Magaña *et al*. [118] and Holzscheck *et al*. [119] both utilized deep learning techniques to develop transcriptomic clocks for predicting biological age. The former applied their models to prefrontal cortex samples, which outperformed traditional methods and identified gene networks involved in signal transduction. This provides insights into transcriptomic changes associated with aging and psychiatric disorders. The latter demonstrated a strong correlation between transcriptomic age and visual age estimates in skin samples. Key aging-related pathways, such as p53- and TNFa/NFkB-signaling, were identified, with *in silico* gene knockdowns validating known aging mechanisms and suggesting new targets for interventions.

In a related effort, Zarrella and Tsurumi [120] analyzed transcriptomic changes in the prefrontal cortex during healthy aging, identifying differentially expressed genes like CA4 and OLFM1. Their models also highlighted genes such as ASPHD2 and CDC42 as important predictors of aging. Qiu *et al*. [121] further advanced aging research by introducing the ENABL Age framework. Their framework enhanced the interpretability of biological age predictions through explainable AI, identifying specific biomarkers and validating their models using large datasets.

In proteomics, Oh *et al*. [122] used plasma proteomics and machine learning to track organ-specific aging, identifying signatures linked to diseases like heart failure and AD, providing practical tools for predicting age-related health outcomes. Wingo *et al*. [123] also explored cognitive aging through proteomic analysis, identifying $579$ proteins associated with cognitive function, offering potential new targets for cognitive aging research.

### How brain age relates to other biological age measures

Alongside brain age, there are various measures such as DNAm age and heart age to estimate the biological age. Exploring the relationships between these measures may reveal distinct aspects of understanding the aging process and its effects on health.

Solovev *et al*. [124] used multi-omics data, including the methylome, proteome, transcriptome, and metabolome data, to estimate biological age. Although their approach helped uncover the complexity of aging and improve predictive models, it lacks the link to brain age. Studies have shown that while DNAm age strongly correlates with chronological age, it may not always align perfectly with BAE, particularly in contexts like neurodegenerative diseases or cognitive decline [125]. Cole *et al*. [5] reported that DNAm age and brain age capture different aspects of the aging process. In their study, combining both measures provided a more robust predictor of aging. The study also introduced the concept of an “aging mosaic,” referring to the variability in aging rates across different biological systems, driven by genetic and environmental factors. Sugden *et al*. [126] emphasized that these two age measures offer complementary insights into aging, particularly with respect to cognitive decline and AD risk. This finding is further supported by Sanders *et al*. [109], who demonstrated that both aging markers capture different aspects of biological processes, particularly in the early stages of life.

To gain further insights into the aging process, Iakunchykova *et al*. [127] investigated the relationship between brain age and heart age. Using deep neural network (DNN), they demonstrated a correlation between heart age, brain age, and cognitive function. This finding highlights the systemic connections between cardiovascular health and brain aging.

In summary, while various measures of biological age, such as DNAm age and heart age, provide insights into different aspects of the aging process, research exploring the relationships among these measures is still limited. Current studies suggest that combining brain age with other measures may offer a more comprehensive understanding of aging, though these measures often capture distinct biological processes.

## Limitations to BAE

**Figure 3:**
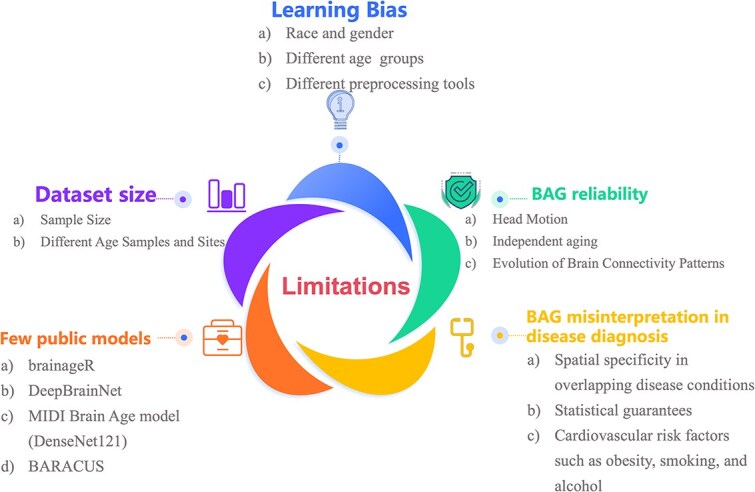
Limitations in current BAE methods.

Recently, the adoption of various deep learning methods and the deployment of localized age predictions have gained attention in studies of BAE. However, the progress in BAE research remains hindered by challenges such as learning biases and limited training data. These challenges are summarized in Fig. 3 and discussed in detail in this section.

### Learning bias

Recent studies have shown significant differences in interpreting BAG across different age stages, races, and genders. Picarra and Glocker [128] demonstrated that common BAE models exhibit significant performance differences across different racial and gender groups, highlighting potential biases in race and gender.

BAE also faces other challenges, including systematic biases such as overestimation in younger individuals and underestimation in older individuals, as well as biases introduced by preprocessing tools. Wang *et al*. [129] identified these common biases and attributed them to the uneven distribution of training data, which causes models to perform inconsistently across different age groups. Jirsaraie *et al*. [83] highlighted that the choice of preprocessing methods and the diversity of training data significantly affect the accuracy and generalizability of age predictions, with different scanner protocols introducing additional errors. Dular *et al*. [130] emphasized that extensive T1-w MRI preprocessing can reduce prediction errors, but the selection and application of these tools can introduce variability. Lu *et al*. [131] pointed out the “regression toward the mean” effect in BAE, where young brains are overestimated and older brains are underestimated, largely influenced by preprocessing choices and dataset selection. Tian *et al*. [132] suggested that the deterioration of physical health in neuropsychiatric disorders often masks changes in brain health, indicating that preprocessing steps might introduce errors affecting the reliability of BAE.

### BAG reliability

Moqadam *et al*. [133] found that head motion during MRI scanning significantly impacts brain age estimates, presenting a major confounding factor that complicates accurate brain age evaluation. Research by Sinha and Raamana [134] suggests that different brain regions undergo independent aging transformations due to neurobiological processes, making it potentially erroneous to simplify high-dimensional imaging features into a single value for assessing brain health. Additionally, Abrol *et al*. [135] highlighted that brain connectivity patterns evolve significantly over time, suggesting that BAGs may vary by age stage. In children and adolescents, high values of BAG might be associated with better cognitive development, indicating a beneficial aspect [136, 137]. However, in middle-aged and older adults, BAGs typically signal accelerated aging and cognitive decline, reflecting poorer health outcomes [138, 139]. These findings underscore the need for age-specific contexts when interpreting BAGs. Without considering these limitations, there is a risk of misinterpreting brain age data, leading to inaccurate assessments of an individual’s cognitive health and developmental progress. Collectively, these studies emphasize the importance of accounting for factors such as head motion, regional brain aging, and age-specific differences to ensure reliability in BAE.

### BAG misinterpretation in disease diagnosis

Recent studies have highlighted several limitations in using BAE for diagnosing psychiatric and neurological disorders. Wei *et al*. [140] found that patients with neuromyelitis optica spectrum disorder and MS exhibited significantly higher BAG values compared with HCs, suggesting that pathological overlaps in these diseases complicate the interpretation of brain age differences. Similarly, Forrest and Kovacs [141] reviewed mixed pathologies in neurodegenerative diseases involving tau protein, TDP-43, A$\beta $, and $\alpha $-synuclein deposits, which further obscure accurate BAG interpretation. Sihag *et al*. [142] addressed this challenge by proposing an explainable BAE framework using covariance neural networks and cortical thickness features, emphasizing the need to account for spatial specificity in overlapping disease conditions.

In addition to pathology-related complexities, comorbidities and broader health factors must be considered when interpreting BAG values. Mouches *et al*. [143] demonstrated that cardiovascular risk factors, such as obesity, smoking, and alcohol consumption, significantly affect BAGs, complicating the interpretation due to overlapping health conditions. Leonardsen *et al*. [91] identified eight genomic regions associated with BAG, revealing that BAE is influenced by a range of genetic and non-genetic traits. Saleem *et al*. [144] noted that while deep learning techniques show promise in diagnosing AD, BAE alone is insufficient for comprehensive diagnosis; additional biomarkers and datasets are necessary. Baldeiras *et al*. [145] echoed this sentiment, warning that relying solely on BAE may result in inaccuracies, especially in differentiating Alzheimer’s from other forms of dementia.

To improve the reliability of BAE in clinical contexts, researchers have explored integrating it with other diagnostic approaches. Tian *et al*. [132] suggested combining BAE with broader assessments of brain-body health to enhance diagnostic accuracy in neuropsychiatric disorders. Ernsting *et al*. [146] proposed a method using uncertainty-aware DNNs and conformal prediction theory to provide statistical guarantees for individual cases, though they raised concerns about the clinical generalizability of their findings, as their study relied primarily on the NAKO dataset, which predominantly includes German subjects and may encompass comorbidities.

In summary, relying solely on BAE increases the risk of misinterpreting BAG results and inaccurately assessing an individual’s cognitive health and disease progression. Therefore, BAE studies should account for confounding factors such as head motion, overlapping pathologies, genetic influences, and comorbid conditions when used for disease diagnosis.

### Few public models

The brainageR, DeepBrainNet, MIDI Brain Age model (DenseNet121), and BARACUS have demonstrated significant utility in predicting brain age. The brainageR uses GPR for predicting the brain age, validating its reliability and predictive capability [147]. DeepBrainNet constructs a deep brain network using large-scale MRI datasets, achieving robust brain age estimates across different scanners and populations [70]. The MIDI Brain Age model (DenseNet121) excels in brain tumor classification, particularly on smaller datasets [148]. BARACUS combines multimodal imaging data to capture cognitive impairment and brain age differences [149]. Despite their impressive performance in research settings, there are very few other publicly accessible models available for direct application. This limitation hinders their broader adoption in clinical and practical applications.

### Dataset size

De Lange *et al*. [48] evaluated the impact of different age ranges and sample sizes on the performance of BAE models, finding that increasing sample size can improve model performance metrics. The study pointed out that insufficient sample sizes can lead to increased prediction errors and affect model stability. This was also supported in another study [47]. Jirsaraie *et al*. [83] studied the generalizability of two brain age models across different age samples, discovering that insufficient sample size and diversity can lead to inconsistent performance across different acquisition protocols, impacting prediction accuracy and reliability. Yu *et al*. [150] systematically evaluated the effects of site harmonization, age range, and sample size on estimating brain age, finding that model accuracy plateaued with sample sizes exceeding $1600$ participants. The study noted that insufficient sample sizes limit model generalizability and stability. Barbano *et al*. [151] investigated BAE using contrastive learning on multi-site datasets, finding that inadequate dataset sizes can lead to models overfitting site-specific noise, thus affecting prediction accuracy and stability. To overcome the limitation of training data, Mateus *et al*. [152] evaluated the feasibility of federated learning for BAE, a method that allows training global models on distributed data to protect patient privacy. However, the effectiveness of this approach still requires further investigation. Overall, these studies consistently demonstrate that increasing sample size is crucial for enhancing the performance and stability of BAE models, while insufficient sample sizes significantly affect model generalizability and prediction reliability.

## Conclusion

Over recent years, brain imaging data have been used more frequently in BAE studies. Although the studies have demonstrated the benefits of using BAG as a biomarker for brain health and clinical diagnosis, the lack of specificity presents a major challenge. This drew the attention of recent studies to explore the effectiveness of local BAE and develop methods for bias correction. Adopting foundation models and deploying federated learning may also enhance the performance of BAE models. However, these directions require further studies. Finally, the clinical applicability of BAE models can significantly expand by integration with other age biomarkers, such as DNAm.

**Key Points**

- Brain age estimation (BAE) using machine learning methods can be beneficial in monitoring brain health and early disease screening.
- Unlike global BAE methods that summarize the whole brain image into a single value (BAG), local BAE methods are advantageous in predicting aging rates in multiple regions.
- Training data, data distribution, evaluation metrics, and gender can significantly influence BAE performance.
- Adopting foundation models and federated learning may pave the way to overcome the significant challenges in BAE studies.
